# Psychodynamic Motivation and Training program (PMT) for the secondary prevention in patients with stable coronary heart disease: study protocol for a randomized controlled trial of feasibility and effects

**DOI:** 10.1186/1745-6215-14-314

**Published:** 2013-09-25

**Authors:** Matthias Michal, Perikles Simon, Tommaso Gori, Jochem König, Philipp S Wild, Jörg Wiltink, Suzan Tug, Björn Sterzing, Josef Unterrainer, Thomas Münzel, Manfred E Beutel

**Affiliations:** 1 Department of Psychosomatic Medicine and Psychotherapy, University Medical Center of the Johannes Gutenberg University Mainz, Untere Zahlbacher Strasse 8, Mainz 55131, Germany; 2 Department of Sports Medicine, Rehabilitation and Disease Prevention, Institute for Sport Sciences, Johannes Gutenberg University Mainz, Mainz, Germany; 3 Department of Medicine II, University Medical Center of the Johannes Gutenberg University Mainz, Mainz, Germany; 4 Institute of Medical Biostatistics, Epidemiology & Informatics, University Medical Center of the Johannes Gutenberg-University Mainz, Mainz, Germany; 5 Institute of Medical Psychology and Sociology, University Medical Center of the Johannes Gutenberg University Mainz, Mainz, Germany

**Keywords:** Psychodynamic psychotherapy, Coronary heart disease, Physical activity, Home based exercise training, Secondary prevention, Spiroergometry, Motivation

## Abstract

**Background:**

Nonpharmacological secondary prevention of coronary heart disease is considered a safe and effective measure to substantially reduce mortality. Despite the effectiveness of lifestyle changes, the compliance rate of patients is very low mainly due to psychosocial barriers. Psychotherapeutic approaches that address how persons think about themselves and their behaviors appear to have a significant potential for improving health behavior.

**Methods/design:**

Against this background, our study aims to examine the feasibility and effects of a Psychodynamic Motivation and Training program (PMT) as compared to one session of advice in exercise training (EX) and treatment as usual (TAU). For that purpose, 90 patients with stable coronary heart disease and a physically inactive lifestyle will be randomly assigned to the three groups (each with n = 30). The primary outcome is the change in the individual anaerobic threshold as determined by spiroergometry from baseline to six month follow-up. Secondary endpoints include change in endothelial function, biomarkers of inflammation and oxidative stress, quality of life, symptoms of fatigue, illness perception and feasibility of the treatment approach. We hypothesize that physical fitness will improve more in PMT than in EX and TAU, with PMT and EX more than TAU, and that the effects will be more pronounced for participants with current mental or psychosocial distress.

**Discussion:**

The results of the study will help to determine the effectiveness of a psychodynamic lifestyle change approach and to identify measures for designing specifically tailored interventions to improve compliance with cardiovascular prevention.

**Trial registration:**

ClinicalTrials.gov Identifier: NCT01445808

## Background

Coronary heart disease (CHD) is the leading cause of disability and death in Western societies. The prognosis of established CHD is strongly influenced by lifestyle factors such as smoking, unhealthy diet and physical inactivity. Among those lifestyle risk factors, physical inactivity is the most prevalent. In Europe, less than 50% of the population is involved in regular aerobic leisure-time, and/or occupational physical activity [1]. Regular physical activity and aerobic exercise strongly reduce the risk of fatal and non-fatal coronary events in patients with CHD, buffer psychosocial distress and improve quality of life and symptoms of depression [2-5]. Therefore, targeting physical inactivity is considered a very important non-pharmacological tool for secondary cardiovascular prevention. Accordingly, for persons with stable coronary heart disease the European Heart Association strongly recommends ‘moderate-to-vigorous intensity aerobic exercise training ≥ three times a week and 30 minutes per session’; and ‘sedentary patients should be strongly encouraged to start light-intensity exercise … after adequate exercise-related risk stratification’ [1].

The overall risk reduction by at least three months of aerobic exercise programs is around 30%. The positive effects of aerobic exercise result from an improved ability to use oxygen to derive energy for work [1], decreased myocardial oxygen demands, improvement of myocardial perfusion, antithrombotic effects, anti-inflammatory effects and anti-arrhythmic effects by favorable modulation of autonomic balance and improvement of endothelial function [1,2].

Despite its favorable effects, implementation of physical activity recommendations has been proven highly problematic in daily life [6]. Psychological and socio-economic factors have been identified as important barriers [7-9]: economic disadvantage, overwork, family duties, feeling physically restricted towards or fearful of exercise, lacking knowledge regarding exercise, negative perceptions towards health, lack of motivation and depression.

Psychotherapeutic strategies have been proven as crucial components of lifestyle interventions, because the interventions need to address how persons think about themselves, their behaviors, and life circumstances [10]. The more these essential components were addressed, the more effective the interventions became [10]. The following techniques were especially important [10]: clarification of the goals and motivation of the individual, providing feedback and reinforcement for progress toward goal achievement, establishing peer-based support, strengthening self-monitoring and self-efficacy. Of note, the authors of the scientific statement of the American Heart Association [10] labeled the above strategies as cognitive-behavioral, however, considering current generic models of psychotherapeutic processes these are better described as general psychotherapeutic strategies [11].

Interestingly, the term ‘lifestyle’ was originally coined by Alfred Adler (1870 to 1937), an Austrian medical doctor, psychotherapist and founder of the psychodynamic school of ‘Individual Psychology’. The ‘style of life’ refers to how individuals live their life and how they handle problems and interpersonal relations [12]. The individual lifestyle is determined early in childhood by the individuals’ interpersonal and social experiences. Health behaviors like physical activity, smoking and dietary habits are essential components of the individual lifestyle. The modern use of ‘lifestyle’ sometimes trivializes health behavior as it suggests that an individuals’ lifestyle might be the result of the individuals’ conscious decisions like choosing to buy a certain brand of chocolate. However, this view neglects the importance of biological, social, developmental, environmental, and psychological factors contributing to the individual’s lifestyle [13-16]. Briefly, the effectiveness of psychotherapeutic techniques as compared to mere provision of information demonstrates that mental factors play a significant role for health behavior. There is already consensus that this is true for smoking, which is regarded as an addictive disorder in line with alcohol abuse or alcohol dependence. In similar ways, this may be also true to some degree for eating behavior or physical activity.

The present study aimed to investigate the effectiveness of a psychodynamic approach to health behavior for secondary prevention of cardiovascular disease. To our knowledge, up to date no psychodynamic treatment studies have been explicitly targeting the improvement of physical activity, respectively lifestyle improvement. However, stronger recognition of lifestyle in psychotherapy is an urgent need. Firstly, the detrimental effects of depression on the development and outcome of coronary heart disease are largely mediated by insufficient physical activity and smoking [2,17-19]. Secondly, poor health behavior is suggested to impair treatment outcome of mental disorder itself [19]. Therefore, targeting lifestyle change is considered to be a crucial component of effective treatments [20]. According to our knowledge, the ongoing stepwise psychotherapy intervention for reducing risk in coronary artery disease (SPIRR-CAD, [21]) is the only psychotherapy study, formulating explicitly health behavior as a treatment focus. SPIRR-CAD was designed to treat depression in patients with coronary heart disease. The focus of the psychodynamic group psychotherapy also included health behavior: there is one session of psychoeducation about modifiable cardiovascular risk factors and each session of the group psychotherapy begins with a brief statement of the patient about his feelings and health behavior.

As psychodynamic psychotherapy is widely-used in Western societies, it is desirable to develop psychodynamic interventions suitable for lifestyle improvement. Especially in Germany, family doctors and other specialists are formally trained in the low-threshold provision of basic mental health care and many have also been trained in psychotherapy for medically ill persons according to psychodynamic principles.

This study aims to determine the feasibility of the new treatment approach, to identify possible effects and effective components for designing specifically tailored interventions to improve compliance with cardiovascular prevention, respectively lifestyle change, that may be worth following up in subsequent larger trials.

## Methods/design

### Study center

The multidisciplinary single center randomized trial with three parallel groups is carried out in collaboration among the Departments of Cardiology, Psychosomatic Medicine and Psychotherapy and Sports Medicine at the University Medical Center of the University of Mainz.

### Participants

Patients are eligible to participate if they fulfill the following criteria: age between 18 to 75 years, stable coronary heart disease determined by percutaneous coronary intervention and low physical activity.

Patients are recruited from the Department of Cardiology of the University Medical Center Mainz and private practices of general practitioners and cardiologists, by research advertisement in the local newspapers and announcements on the webpage of the University Medical Center. Further, patients who were hospitalized for percutaneous coronary intervention during the last ten years are actively approached with an information letter. Once a patient articulates his interest in participation, he signs an informed consent allowing the inspection of any medical records and screening for physical activity. The individuals’ medical records are reviewed for the inclusion and exclusion criteria by a research assistant and a medical doctor. Low physical activity was defined as less than 15 to 20 minutes of at least moderate exercise training per week during the last month according to the definition of the Heart and Soul Study [17]. This criterion was a strong predictor of mortality in post-myocardial patients [17]. After the review of the medical records, the patient is contacted, usually by phone, and interviewed for inclusion and exclusion criteria. Further, he receives information about the procedures and aims of the study. If the patient is eligible and willing to participate, he/she is invited to the study center for further information. After discussion of the study procedures, he/she is asked to sign the informed consent for trial participation. Participating patients are paid an allowance of €25 for the baseline assessment and €75 for the follow-up assessment.

Table 1 lists the inclusion and exclusion criteria. Inclusion and exclusion criteria were selected based on the following considerations: firstly, in order to ensure a valid diagnosis of coronary heart disease, a percutaneous coronary intervention was required. Secondly, in order to keep the threshold for participation low. Thirdly, in order to be able to analyze biological outcome parameters such as inflammation, endothelial function and anaerobic threshold. For the latter purposes, the participants should not be treated with insulin or systemic immunosuppressant drugs. Changes in medication with beta-blockers, statins and drugs acting on the renin-angiotensin axis in the last two weeks before each planned visit were not allowed (that is, visits were rescheduled).

**Table 1:** Inclusion and exclusion criteria

	
Inclusion criteria:	
1.	Stable coronary heart disease with CCS functional classification of angina class I to III
2.	Low self-rated physical activity
3.	Condition after percutaneous coronary intervention > 4 weeks until < 26 weeks or > 52 weeks after index percutaneous coronary intervention
4.	Residence < 50 km radius of the study center (city of Mainz)
5.	If treatment with beta blockers or ivabradine then stable > 4 weeks
Exclusion criteria:	
1.	Acute coronary syndrome or myocardial infarction < 8 weeks
2.	Coronary stenosis of the dominant vessel > 25% or high grade stenosis of the left coronary artery
3.	Heart failure with left ventricular ejection fraction < 40%
4.	NYHA III to IV
5.	Severe heart valve disorder
6.	Insulin dependent diabetes
7.	Orthopedic or other disorders, which preclude regular physical activity
8.	Coronary artery bypass surgery < 6 months before index PCI
9.	Severe obesity (BMI ≥ 40)
10.	Need for systemic immunosuppression with cortisone or methotrexate
11.	Kidney failure with need for dialysis
12.	Intake of nitrates < 12 hours

*CCS* severity of angina according to the Canadian Cardiovascular Society, *NYHA* symptoms of heart failure according to the New York Heart Association, *PCI* percutaneous coronary intervention.

### Interventions

The study has three arms, two active arms and the control condition ‘treatment as usual’. The active arms added to the control condition ‘treatment as usual’.

### Control condition

Treatment as usual (TAU) comprises regular treatment by the general practitioner, including standard lifestyle recommendations, cardiological and any other health care utilization by the patient. As in Germany all persons have full insurance coverage for medical care including long-term outpatient psychotherapy, TAU represents ‘optimal medical care’.

### Advice in exercise training

The second arm consists of one session of detailed and individualized advice in exercise training (EX). Counselling by an experienced sport medicine specialist is based on the findings of the present spiroergometry and the review of the medical records. The participant receives an exercise-plan with recommendations of endurance exercise time or resistance training based on guidelines for adults from the American College of Sports Medicine and the American Heart Association [22] and individual heart rate range in two different intensities of training for aerobic exercise activity.

### Psychodynamic motivation and training program

The Psychodynamic Motivation and Training program (PMT) follows a psychodynamic stepped care approach. It consists of two to eight sessions of individual psychodynamic psychotherapy in addition to EX. Session frequency, mode (for example. face-to-face consultation, telephone) and dosage are tailored to the individual needs. The maximum dosage is limited to 400 minutes per patient over six months. The individual need is determined by the regular monitoring of physical activity and the patients’ wishes. PMT is conducted by a psychodynamic psychotherapist (board certification or supervision by certified psychotherapist).

PMT is a psychodynamic stepped treatment approach integrating components that have been proven effective for lifestyle change [10] and stress regulation (for example. mindfulness exercises, [23]). The stance of the psychotherapist is actively engaged, goal-directed and directive as described in current manuals of short term psychodynamic psychotherapy [24]. The therapist is willing to teach and guide the patient. The therapist identifies negative transference issues immediately, so that they can be worked through as soon as they arise. Every effort is done to establish the necessary alliance and trust in the therapeutic relationship. As intellectual insight into the need of improved health behavior and the readiness to change are quite separate issues, the therapist must help the patient to experience the benefits of his adaptive wishes and the costs of his maladaptive behavior emotionally. PMT targets the mental and external barriers to improving physical activity by focusing on affects, interpersonal relations and self- and other representations. Unhealthy behavior is considered as the result of maladaptive self-representations (that is, lack of self-care due to blocked positive feelings toward the self) and dysfunctional capacities of anxiety respectively stress regulation (for example, stressful overeating, smoking, addictive television watching, blocked assertiveness to pursue one’s goals). The intervention aims at motivating the patient to be more caring about himself as reflected in improved health behaviors, especially in a physically more active way of living. Physical activity is defined as daily life activities (walking instead of driving, staircase instead of elevator and so on) and home-based exercise training of moderate or vigorous intensity (for example, jogging, swimming, participating in coronary exercise groups, or supervised endurance and weight training).

The first session starts with the questions ‘What motivated you to participate in this study?’ and ‘What about the recommendations of the sport medicine specialist, how have you succeeded in following them?’. These questions give a good introduction into the discussion of the relevant themes. The aims of the first sessions are to establish a secure therapeutic alliance, to take a bio-psycho-social history, to identify maladaptive health behaviors, assess subjective illness concept and disease knowledge, manner of health care utilization and predominant coping strategies. Further, inner resistance (lack of motivation, hopelessness and so on), lack of knowledge, external barriers (lack of time, financial problems), and resources (partnership, neighborhood, personal strengths) towards realizing a more physically active style of living are explored. On the basis of this dialogue, an individual health plan is designed together with the patient. The health plan follows the structure of ‘My Better Health Plan’ as described in the TEAMcare Intervention Manual [25]. The health plan consists of the goals of the patient, the measures to achieve this goal, a point-to-point to-do-list, explanatory notes on mental barriers towards following the to-do-list and the date of the next visit for monitoring the targets. Table 2 gives an example of a personalized health plan of the first session from the patient Mrs. A of vignette 1 (Additional file 1). To note, for the collaborative design of the ‘Health Plan’ it is not sufficient that the patient formulates his wish to improve his fitness in order to live longer. It is very important that the patient can imagine the benefits of improved health behavior vividly with a sense of positive feelings and hope. These positive emotions need to be labeled and validated. The health plan and a copy of the ‘National Patient Guideline Coronary Heart Disease’ are handed to the patients.

**Table 2:** Health plan

	
My goals	I want to improve my health, my physical and cognitive fitness, in order to preserve my autonomy, because I enjoy living. I want to spend more years with my son and his wife and I am looking forward to become a grandmother and see my grandchild grow up.
The measures to achieve my health goals	1) I need to be more physically active, at least 150 minutes and at best 300 minutes of moderate exercise training per week; 2) I need to quit smoking urgently; 3) I need to care more about myself (for example, meet friends and do things that I enjoy such as traveling and cinema/theater); 4) I need to improve my dietary habits (less alcohol, less chocolate, more fruits and vegetable).
My to-do-list	1) 20 minutes brisk walking at pulse range of 90 to 105 beats per minute every second day; 2) I will reflect on my reasons for smoking and try to become more aware of the harm I cause to the most important goals in my life; 3) I will carefully read the patient guideline for coronary heart disease and reflect about it.
Potential obstacles	I am in danger of missing good opportunities for engaging myself for important goals due to maladaptive feelings of guilt. These guilt feelings block healthy impulses, for example, doing what I like, assertiveness, and taking a long-term perspective on my life.
Check-up	Date of next visit.

Vignette 2 describes a patient Mr. B. with less need for psychotherapeutic support (Additional file 2). During the follow-up sessions the health plan is monitored and individually adapted to the patients’ needs. In each session, internal and external barriers and the individual resources for goal achievement are explored. Concerning physical activity, the aim is to increase the dosage of exercise training to 300 minutes of moderate or 150 minutes of vigorous training per week as recommended by the medical guidelines. In the second session, the reading assignment of ‘National Patient Guideline Coronary Heart Disease’ is reviewed: ‘Did any questions arise? What were your feelings regarding this guideline?’ Sometimes patients report that the reading of the guideline elicited anxieties. This occurs especially in patients who tended to deny that they have a chronic disease. After reading the guideline they recognize that coronary arteriosclerosis is not cured by coronary angioplasty, but lifestyle change and long-term coping strategies are required. The review of the reading assignment is followed by the exploration of medical risk factor control and adequate health care utilization (‘Do you regularly measure your blood pressure? Is your blood pressure below 140/90 mmHg?’; ‘Do you know your cholesterol levels? Is your LDL below 100 mg/dl?’). If problems are identified the patient is encouraged to consult his general practitioner for engaging in target control.

In the second or third session, an offer is also made to the patient to include his/her partner into treatment with the purposes of information and improvement of social support for establishing a healthy style of life. In addition to the treatment targets physical activity and health behavior, the patient is encouraged to speak about all other issues that bother him. These psychosocial issues concern mainly difficulties with the partner, stress at work or stress of taking care for family members, and difficulties with communicating their needs to their doctors.

If a mental disorder is identified during the course of the treatment, the patient is informed about treatment options according to the current medical guidelines. Psychopharmacotherapy may be initiated during the trial. If there is need for further psychotherapy, the patient will receive specific recommendations and information how to obtain this.

### Assessment

**Figure 1:**
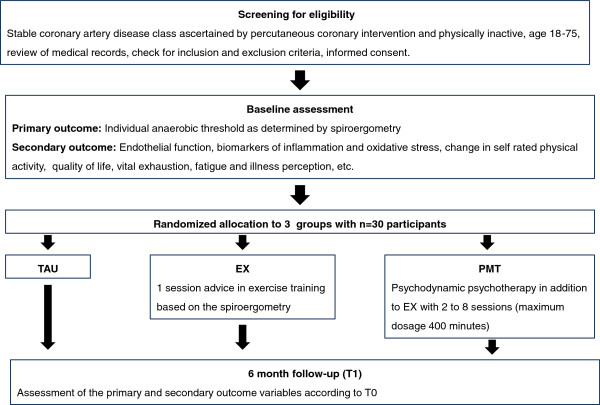
Study design.

The assessment includes spiroergometry, blood and urine sampling, measurement of endothelial function and psychometric questionnaires at baseline (T0) and six months follow-up (T1). Spiroergometry, anthropometry and blood sampling are conducted in the Department of Sport Medicine of the University Mainz. Measurement of endothelial function, blood pressure measurement, blood and urine sampling are performed in the Department of Cardiology. Assessors are blinded to the allocation of the patients. Medical diseases and medications are captured by the review of recent medical records and patient interview. Figure 1 gives an overview of the study design and time points of assessment.

### Spiroergometry

The spiroergometry is performed on a bicycle ergometer according to a standardized protocol. Ergometry is carried out in supine position on a computer-controlled bicycle ergometer (Ergoselect 200P, ergoline GmbH, Bitz, Germany). The ergometry protocol starts at 20, 25 or 50 Watts depending on the expected maximal work load and is increased by 20 or 25 Watts every three minutes. The patients are instructed to keep a pedal rate of 60 rpm. A 12-lead electrocardiograph (ECG) is continuously recorded. Non-invasive blood pressure is measured before exercise, at the beginning and end of every stage, and three and six minutes after exercise. Heart rate is registered continuously by ECG. Oxygen uptake (VO_2_), CO_2_ output (VCO_2_), and minute ventilation (VE), are measured with an open system spirometer (Ergostick, Geratherm Respiratory, Bad Kissingen, Germany). The respiratory parameters are measured breath-by-breath, starting one minute before and ending six minutes after exercise. Breath-by-breath data are averaged over consecutive intervals of three seconds. Patients are advised to cycle until complete exhaustion. Exercising is stopped if one of the following symptoms occurs: chest pain, ST-segment depression, ventricular arrhythmias, systolic blood pressure > 250 mmHg or diastolic blood pressure > 115 mmHg, or a drop in blood pressure.

Lactate is measured at rest and following each workload stage and three and six minutes after exercise by taking capillary blood samples from the hyperemic earlobe. The individual anaerobic threshold (IAT) is determined from the lactate curve by determination of the relative work load (Watt/kg) when a 1 mmol rise in lactate can be found above the minimum lactate equivalent [26,27]. The ventilatory threshold (VT) is determined by analysis of the increase in the respiratory equivalent for oxygen or by decrease in the respiratory equivalent for carbon dioxide [28]. The V-slope method [29] and typical changes in end-tidal gas concentrations (PETO_2_ and PETCO_2_) are examined to search for agreement to control for precision of VT determination.

### Endothelial function

Endothelial function is a valid surrogate marker for cardiovascular disease. The extent of endothelial dysfunction appears to reflect the traditional risk factor burden [30]. The methods employed for the analysis of endothelial function using flow-mediated dilation and constriction (FMD and L-FMC) have been previously published [31]. Briefly, L-FMC and FMD are measured using a Vivid 7 (General Electrics, Munich, Germany) ultrasound platform equipped with a 14 MHz matrix probe and a micrometric probe holder. L-FMC is defined as the constriction observed in the last 30 seconds of a 4.5 minute occlusion of a pneumatic cuff placed distal to the site of arterial diameter measurement and provides information on resting endothelial function. FMD is measured as the maximal dilation observed in the five minutes following deflation of the cuff, that is, during reactive hyperemia, and corresponds to endothelial responsiveness or recruitability. Both markers have been shown to be blunted in the setting of coronary artery disease, hypertension, heart failure, and after smoking [32]. Arterial diameter data are acquired digitally and analyzed in a randomized, blinded fashion using automatic dedicated software whose repeatability and reproducibility has been recently reported [33]. Flows will be analyzed from the digitized audio data using the automated software built in the ultrasound machine.

### Blood pressure measurement

Blood pressure will be assessed by a trained research assistant (blinded to treatment), using the auscultatory method, with a calibrated sphygmomanometer and stethoscope.

### Anthropometry

Anthropometry includes assessment of height and weight, waist-to-hip ratio (WHR) and determination of body composition (body fat, muscle mass, intracellular and extracellular water) by the InBody 3.0 system (Biospace Ltd. 1999) 518–10 Dogok 2-dong, Gangnam-gu, Seoul, 135–854 Korea; http://www.e-inbody.com/.

### Blood and urine sampling

Humoral markers will be obtained from fasting blood samples. Serum lipid levels (total cholesterol, triglycerides, and high-density lipoprotein cholesterol), plasma levels of C-reactive protein, fibrinogen, albumin levels and HbA1c are measured immediately after blood withdrawal by routine methods; low-density lipoprotein cholesterol will be calculated by the Friedewald formula. Markers of oxidative and inflammatory stress will be determined in plasma or serum stored immediately after blood withdrawal and centrifugation at −80°C until analysis. The measurements will be done in a blinded fashion in a single batch.

### Psychometric questionnaires

Quality of life is assessed by the EQ-5D™ (EuroQuol Group, Rotterdam, Netherlands) [34]. The EQ-5D™ captures problems pertaining to usual activities and anxiety/depression dimensions, and the respondent’s own assessment of their health status on a visual scale from 0 (worst imaginable health) to 100 (best imaginable health). Coronary artery disease related quality of life is measured with the Seattle Angina Questionnaire (SAQ) [35].

Feelings of vital exhaustion are assessed by the Maastricht Questionnaire [36] and symptoms of fatigue by the MFI Multidimensional Fatigue Inventory (MFI) [37].

Insomnia symptoms were measured by the Jenkins sleep questionnaire [38], covering difficulties initiating and maintaining sleep as well as non-restorative sleep. Occurrence of sleep disturbances is rated for the previous four weeks from: not at all (1), 1 to 3 days (2), 4 to 7 days (3), 8 to 14 days (4), 15 to 21 days (5) to 22 to 28 days (6).

The cognitive and emotional representations of the coronary heart disease are measured by the brief Illness Perception Questionnaire (IPQ) [39]. Changing patients’ illness perceptions may improve outcome in coronary heart disease [40].

Self-rated habitual physical activity was measured by the Habitual Physical Activity Questionnaire [41] and the one item screener from the Heart and Soul Study [17]: ‘Which of the following statements best describes how physically active you have been during the last month, that is, done activities such as 15 to 20 minutes of brisk walking, swimming, general conditioning, or recreational sports?’ Participants chose from one of the following six categories: not at all active, a little active (1 to 2 times per month), fairly active (3 to 4 times per month), quite active (1 to 2 times per week), very active (3 to 4 times per week), or extremely active (≥ 5 times per week). Being ‘not at all’ or ‘a little’ active is considered as physically inactive.

Different dimensions of mental distress are assessed by the Patient Health Questionnaire (PHQ) [42]: depression, anxiety, somatic symptoms and ten common psychosocial stressors. The depression module comprises nine symptoms of depression (PHQ-9). The scores range from 0 to 27. A cut-off ≥ 10 yields a sensitivity of 81% and a specificity of 82% for detecting any depressive disorder [43]. Anxiety is assessed by the Generalized Anxiety Disorder Scale (GAD-7) [44]. Scores range from 0 to 21 with higher values reflecting more anxiety. Somatic symptoms severity is assessed with the 15 items of the PHQ-15. Scores range between 0 to 30. Scores above 15 identify individuals with high levels of somatic symptom severity respectively somatization severity [45]. In addition to symptoms of mental disorders, the PHQ assesses concerns about ten major psychosocial stressors (for example, financial status, family relationships, work, health) on a three-point scale (not bothered, bothered a little, bothered a lot) [42]. For the determination of a subgroup of persons with increased mental or psychosocial distress, the following scores will be calculated: item #3 of the PHQ-9 will be used as a screener for sleep disturbances as a previous study found that the sleep disturbance item correlated strongly with the well-validated Insomnia Severity Index [46]. Sleep disturbances more than half the days will be determined as significant. Clinically significant anxiety will be determined by the two-item version of the GAD-7 [47] with a cut-off score of three or more [48]. The two-item depression module of the Patient Health Questionnaire (PHQ-2) will be calculated also [47]. As in a previous study a PHQ-2 score of two or more predicted the mortality of patients who underwent PCI, we applied this cut-off [49].

### Cognitive functioning

After having started the recruitment for the study we decided to include a computer based version of the Tower of London - Freiburger Version [50] in order to identify changes of cognitive functions. The Tower of London (ToL) is a frequently used neuropsychological test instrument for assessing planning ability in various clinical and healthy populations [51]. In its original version, three balls of different colors are placed on three different rods of different lengths [52] and subjects are presented with a start state and instructed to transform it into a given goal state. In order to solve the problem in the least possible number of moves, subjects are thus requested to plan ahead a solution before manually executing the moves. Three rules have to be followed: (i) only one ball can be moved at a time, (ii) balls must not be placed outside the tower, and (iii) if more than one ball is stacked on a rod, only the topmost ball can be moved.

The ToL problem-set applied in the study consists of an optimized problem selection using eight four-, five-, and six-move problems each of which instantiate a linear increase of problem difficulty [53]. Test duration was limited to a maximum of 20 minutes. The test has been proven responsive to exercise interventions [54,55]. We estimate that 30 complete measurements will be available at study finalization.

### Objectives and hypotheses

The purpose of this study is to examine the effectiveness of the Psychodynamic Motivation and Training program (PMT) for the improvement of physical fitness as determined by the individual anaerobic threshold in patients with stable coronary heart disease as compared to one session of advice in exercise training (EX) only, or treatment as usual (TAU). We have the following hypotheses:

1) PMT is more effective than EX only and TAU concerning change in individual anaerobic threshold.

2) The advantage of PMT will be more pronounced for persons with current mental distress and psychosocial stressors as compared to persons without such stressors.

3) Both active interventions (PMT, EX) are more effective than TAU concerning change in individual anaerobic threshold and secondary endpoints.

4) PMT will be more effective in improving illness perception, quality of life and symptoms of psychosocial distress than EX or TAU.

### Outcomes

The outcomes are determined by change from baseline to six month follow-up. The primary outcome is change in power (Watt/kg) at the individual anaerobic threshold according to lactate kinetics during spiroergometry. Although the maximal oxygen uptake (VO_2−_peak) is currently the most popular risk stratification factor for patients with coronary heart disease, we decided to determine the individual anaerobic threshold as our primary outcome. It is well known that assessment of VO_2−_peak is limited by the ability of patients to engage in maximal exercising and influenced by their motivation and perceived exertion. Therefore, submaximal reference values of exercise capacity, like oxygen uptake at the ventilatory threshold (VT), have been demonstrated to be more objective [56]. Submaximal exercise values such as VT and ventilatory efficiency have been described as better predictors for mortality or major cardiac events than VO_2_-peak [57,58]. The concept of the lactate threshold for the determination of training loads is a method for endurance performance assessment and for the prescription of exercise intensities that is valid and reliable [59]. Particularly, individual anaerobic thresholds (IAT) determined by lactate kinetics in graded exercise treadmill protocols have been described to have an even higher test-retest reliability then the determination of the ventilator threshold [26].

More recently, a two-month residential rehabilitation program, including exercise training in patients with chronic heart failure, revealed that all exercise intensity related values at the IAT including VO_2_, VCO_2_, work rate in Watts, and VE were much more significantly improved than the respective values at maximal exertion [60]. We therefore decided to determine the IAT as main outcome variable in our intervention using a graded exercise protocol as developed by Dickhuth and co-workers [26] that is adjusted and validated for cycling [27].

Secondary outcomes are change in maximal aerobic capacity (VO_2−_peak); change in endothelial function according to the flow mediated dilatation; change in high-density lipoprotein levels; change in biomarkers of inflammation and oxidative burst; change in the waist-to-hip ratio and body mass index (BMI kg/m^2^) as determined by supervised medical anthropometry; change in the self-rated habitual physical activity according to the Habitual Physical Activity Questionnaire; change in quality of life according to the visual analog scale of the EQ-5D; change in the severity of impairment by angina symptoms according to the Seattle Angina Questionnaire; change in fatigue according to the Maastricht Questionnaire; change in quality of sleep according to the Jenkins Sleep Questionnaire; and change in illness perception according to specific items from the brief Illness Perception Questionnaire (item #1 ‘consequences score’; item # 3 ‘personal control’ and item # 6 ‘illness concern’). In a subgroup of participants, we will further be able to explore change in performance of goal planning according to the ToL.

Feasibility of the intervention will be assessed by analyzing procedures for recruitment (ratio of patients screened versus included), acceptability of allocation procedures and attrition rates (loss to follow-up measurements, completion rates) between the three conditions. Further, the dosage of PMT will be described by minutes and mode of intervention.

### Sample size calculation

As this is the first study on the effectiveness of a psychodynamic motivation and training program, we designed the study primarily to test the feasibility of PMT and possible effects that may be worth following up in a subsequent larger study. The sample size calculation was based on the following considerations: in general, exercise programs for patients with CHD yield increases in the aerobic capacity as determined by the VO_2_-peak of 2.6 ± 1.6 (mean ± standard deviation) versus 0.3 ± 1.4 mL/kg/min in the inactive control groups [61]. This difference corresponds to an effect size of about 1.5 standard deviations. With regard to change of the anaerobic threshold, a recent study on the effects of exercise training in patients with chronic heart failure found at the lactate threshold, a mean workload difference of 43 Watts with a standard deviation of about 17 Watts [60]. For our study we estimated a 50% lower effect size differences, as the exercise training was not supervised and therefore presumably less intensive.

Comparing PMT to EX and to TAU, while controlling the multiple type one error at a level of 0.05, each comparison has to be performed at the nominal level of 0.025 using the Bonferroni correction. With a sample size of n = 30 in each group, a standardized effect size of 0.82 can be detected at the two-sided level 0.025 with a power of 0.80. Assuming that the standard deviation of change in the individual anaerobic threshold is 17 Watts (which is roughly in accordance with results from [60]), this corresponds to an absolute effect of 14 Watts for PMT, which is less than half of the effect reported in [60].

### Randomization

The participants are randomized to three groups with n = 30 patients each. A computer program randomly assigned numbers from 1 to 90 to three sets representing the three groups. The numbers were hidden in closed envelopes. For each new participant an envelope was opened and the participant was allocated accordingly.

### Statistical methods

Categorical variables are presented as frequencies and percentages (n,%), continuous variables as means and standard deviations (mean ± standard deviation), or medians and quartiles for variables with skewed distributions (median (25th quartile, 75th quartile). Baseline characteristics of the three treatment groups will be compared with respect to age, sex, socioeconomic status, partnership, cardiovascular risk-factors (for example, smoking, obesity), comorbidity, variables characterizing patients with coronary heart disease (for example, medication) and outcome parameters.

Primary and secondary endpoints will be analyzed according to the intention-to-treat principle. Change from baseline to follow-up will be analyzed by analysis of covariance adjusting for baseline measurement of the respective outcome. Doing so, separate contrast are specified for comparing PMT with EX and TAU, respectively. Cohen‘s effect size will be calculated. In order to determine whether the effect of PMT is modified by presence of significant mental distress and psychosocial stressors at baseline, a test for interaction will be performed. Exposure to significant mental distress and psychosocial stressors will be defined by at least one of the following baseline criteria: PHQ-2 ≥ 2, GAD-2 ≥ 3, sleep disturbances (according to item #3 of the PHQ-9 ≥ ‘more than half the days’), and common psychosocial stressors according to the PHQ module (‘being bothered a lot’ by ‘difficulties with husband/wife, partner/lover or boyfriend/girlfriend’, or ‘stress of taking care of children, parents, or other family members’, or ‘stress at work outside of the home or at school’, or ‘having no one to turn to when you have a problem’). All analyses will be conducted on a two-sided level of significance with *P* < 0.05.

### Ethical issues

The final study protocol and the final version of the written informed consent form were approved by the Ethics Committee of the Federal State of Rhineland Palatine in Germany (ref: 837.274.11.7816). The procedure set out in this protocol, pertaining to the conduct, evaluation, and documentation of this trial, were designed to ensure that all persons involved in the trial abide by the code of Good Clinical Practice and the ethical principles described in the current revision of the Declaration of Helsinki. The trial will be carried out in keeping with local legal and regulatory requirements. Before being admitted to the clinical trial, patients must consent to participate after the nature, scope, and possible consequences of the clinical trial have been explained in a form understandable to them. The patients must give written informed consent to participate in the study, including their consent to publish. Any findings during the study assessments that need further clarification are communicated to the patient by a letter with specific recommendations. For example, if there are clinically significant depressive symptoms, the patient will be informed about this finding with the recommendation to consult his general practitioner for further diagnostic evaluation and treatment. The participating patients are paid an allowance of €25 for baseline assessment and €75 for the follow-up assessment.

## Discussion

According to our knowledge, this is the first study investigating effects of psychodynamic psychotherapy on cardiorespiratory and physical fitness. We hope that the study will yield knowledge about the optimal dosage of intervention an individual patient needs, respectively which patients profit from such individualized treatment approaches, and for who personalized exercise advice or even treatment as usual might be sufficient. As this study focused primarily on motivational barriers for lifestyle improvement, no supervised exercise training was applied. Home based exercise training was considered as the appropriate mode, as this keeps the threshold for participation lower as opposed to structured supervised exercise training with regular schedules. Important strengths of the study are the use of biological outcome parameters. Changes in the individual anaerobic lactate threshold and oxygen consumption are much more reliable measures of improved physical activity than self-reported increases in physical activity. Further, by analyzing change of endothelial function we will be able to describe effects on an important surrogate parameter of cardiovascular disease.

A major limitation of the study is the short duration of observation. The investigation of the sustainability of exercise and life style compliance needs at least one or more years of observation. Another important limitation concerns the recruitment of participants. As the participants need to give their informed consent to participate in this trial, we expect that the vast majority of the patients are already at the stage of contemplation about the necessity to improve their physical activity. This is to say, we will not be able to recruit pre-contemplative patients [62,63]. These latter pre-contemplative patients, however, will presumably have the most severe lifestyle problems as they are even not emotionally aware of the detrimental consequences of their behavior. These patients actually represent a target population of our psychodynamic approach, as psychotherapy especially helps to build up motivation, for example, by helping to experience emotionally the costs of one’s maladaptive behavior. Due to this selection-bias, inferences from our study to pre-contemplative patients will be restricted. Trial designs have to be developed that enable both informed consent and inclusion of ‘unwilling’ patients. Further limitations comprise the lack of a more elaborated assessment of blood pressure (for example, home or ambulatory blood pressure measurements at regular intervals). Due to this lack, the analysis of effects on high blood pressure is limited. However, blood pressure at baseline and follow-up will be considered as a potential confounder of the endpoints. If adequate, additional adjustment for blood pressure will be applied. Another limitation is the lack of the assessment of endothelium-independent vasodilatation [64-66]. However, a number of studies showed no effect of exercise on nitroglycerin (endothelium-independent) responses, at least in the forearm circulation, and that these responses were not associated with VO_2−_peak levels [67]. Last but not least, differences in the initiation of psychopharmacotherapy may constitute a potential bias regarding secondary endpoints such as fatigue and quality of life. In order to be able to control for this potential bias, the rate of psychopharmacotherapy will be assessed at baseline (current) and at follow-up (past, current).

## Trial status

Recruitment of participants is ongoing. The first participant was included in October 2011. We expect that the last patient will have finished by May 2014.

## Abbreviations

BMI: Body mass index; CCS: Severity of angina according to the Canadian Cardiovascular Society; CHD: Coronary heart disease; CRF: Cardiovascular risk-factors; ECG: Electrocardiography; EX: Advice in exercise training; FMD: Flow-mediated dilation; GAD-2: Two-item anxiety version of the GAD-7; GAD-7: Generalized anxiety disorder scale; HDL: High-density lipoprotein; HPAQ: Habitual physical activity questionnaire; IAT: Individual anaerobic threshold; IPQ: Brief illness perception questionnaire; LDL: Low-density lipoprotein; L-FMC: Flow-mediated constriction; MFI: Multidimensional fatigue inventory; NYHA: Symptoms of heart failure according to the New York Heart Association; PCI: Percutaneous coronary intervention; PHQ: Patient health questionnaire; PHQ-2: Two-item depression module of the PHQ; PMT: Psychodynamic motivation and training program; SAQ: Seattle Angina questionnaire; TAU: Treatment as usual; ToL: Tower of London; VE: Minute ventilation; VO2-peak: Maximal oxygen uptake; VT: Ventilatory threshold; WHR: Waist-to-hip ratio

## Competing interests

The authors declare that they have no competing interests.

## Authors’ contributions

MM, MEB, TG, PS, JU, JK, ST prepared the first draft of the manuscript. MM, MEB, TG, PS, JW, JK, JU, PSW, TM revised the final draft of the manuscript and critically revised it for its intellectual content. MM, MEB, PS, TG, JK, ST, BS and TM substantially contributed to the conception and the design of the study. All authors read and approved the final manuscript.

## Supplementary Material

Additional file 1 Vignette 1; psychodynamic case report (Mrs. A.). Click here for file

Additional file 2 Vignette 2; psychodynamic case report (Mr. B.). Click here for file
