# Supplementary material for: Psychodynamic Motivation and Training program (PMT) for the secondary prevention in patients with stable coronary heart disease: study protocol for a randomized controlled trial of feasibility and effects
Source: Trials. 2013 Sep 25;14:314. doi: 10.1186/1745-6215-14-314 (PMC3819661; doi:10.1186/1745-6215-14-314)
Supplement: Additional file 1 — Vignette 1; psychodynamic case report (Mrs. A.). [file 1745-6215-14-314-S1.doc]

**Vignette 1**

**Patient:** Mrs. A., age 66, retired nurse (since 1 year), married, husband handicapped by a stroke, one married son, no grandchildren

**Medical history:** 2 vessel coronary heart disease since 7 years, last PCI 2 years ago, gastric reflux disease, spinal disc herniation 6 years ago, currently moderate back pain, no history of a previous mental disorder

**CRF**: Smoking 10 cigarettes/d, sedentary lifestyle since retirement, overweight (BMI 28)

**Psychosocial findings in the patient health questionnaire:** Mild depressive symptoms in the PHQ-9 (score = 6) corresponding to subthreshold depression, bothered a lot by difficulties with the partner and lack of social support (“having no one to turn to”).

**Psychodynamic case report:** At the beginning of the 7 session treatment, Mrs. A. was already aware of her unhealthy behavior. However, guilt feelings and a tendency to deny the detrimental effects of her behavior on her life impeded change on the one hand. On the other hand, she articulated the wish to improve her health in order to be able to enjoy life (travel) and take part in the life of her son and her desired grandchildren in the future. A major source of distress was the stroke of her 8 year elder husband. Since his stroke, 2 years ago, her husband has fallen into inactivity and reticence, spending the whole day on the couch watching TV and drinking abusively alcohol. Medical help was actively refused by him. In this situation Mrs. A was handicapped by guilt feelings in pursuing her goals. The treatment helped her to find more adaptive ways of dealing with her husband. To her surprise, he responded understandingly when she started with her exercise training. Later, she also joined a yoga group and started to meet her friends for cinema visits or excursions. Smoking was identified as a dysfunctional measure of calming down and feeling free. Taking a cigarette break on the balcony provided her the distance needed to her husband and to related distressing feelings. During therapy she quitted smoking. Instead, she started mindful breathing regularly and used it as an emergency measure when distressing feelings came up (e.g. deep breathing on the balcony instead of taking a cigarette break). At the end, she reviewed that PMT had helped her to take a different point of view. She felt less helpless, physically and mentally stronger and could cope better with her marital situation. Even though the situation of her husband did not improve, more acceptance and even humor was established between them. She also managed it better calling for support from her son and friends. The total dosage of therapy was 350 minutes.
