# Supplementary material for: Psychodynamic Motivation and Training program (PMT) for the secondary prevention in patients with stable coronary heart disease: study protocol for a randomized controlled trial of feasibility and effects
Source: Trials. 2013 Sep 25;14:314. doi: 10.1186/1745-6215-14-314 (PMC3819661; doi:10.1186/1745-6215-14-314)
Supplement: Additional file 2 — Vignette 2; psychodynamic case report (Mr. B.). [file 1745-6215-14-314-S2.doc]

**Vignette 2**

**Patient:** Mr. B., age 68, retired clockmaker, widowed since 17 years, living in a new partnership since 10 years, one daughter from his first marriage, two grandchildren

**Medical history:** 2 vessel coronary heart disease since 15 years, coronary bypass graft 15 years ago, last PCI 4 months ago due to non-ST acute myocardial infarction, no history of any previous mental disorder

**CRF**: Low physical activity, hypertension (well controlled)

**Psychosocial findings in the patient health questionnaire:** Worries about his health, mild unspecific chest pain, no significant symptoms of depression or anxiety.

**Psychodynamic case report:** Mr. B entered the study in a state of health concerns due to his recent myocardial infarction. Actually, he was not physically inactive since he walked his dog daily. However, the walking corresponded only to light exercise intensity. Since his recent non-ST acute myocardial infarction, he was worried about his physical capacity. Previous information of his doctors could not give him a sense of security. Due to his non-pathological obsessiveness, which indeed had been a unique resource of success in his career as a clockmaker, it was impossible for him to rely on scarce information such as “no problem, you can jog unless you don’t feel angina”. However, after receiving detailed individualized advice by the sport medicine specialist based on the spiroergometry, he felt more confident and could start engaging in regular exercise training of moderate and increasingly vigorous intensity. Pulse range and pulse watch, and comprehensive information about angina symptoms, gave him a sense of security so that he could increase his physical fitness and confidence significantly. Sessions two and three focused on his communicative behavior towards his doctors, in order to enable him to be more assertive, i.e. not to leave his doctor's office unless he got an adequate answer on his concerns. The treatment dosage was 115 minutes (3 face-to-face sessions of a total amount of 100 minutes and 3 brief telephone sessions).
